# A Temporal Analysis of the Cost of Aesthetic Surgical Procedures Over the Past Decade in Relation to Consumer Price Indices and the Stock Market

**DOI:** 10.1093/asjof/ojad027.007

**Published:** 2023-04-14

**Authors:** Anooj Patel, Pedram Zargari, Karan Chopra

**Affiliations:** University of Minneosta, Minneapolis, MN; University of Minneosta, Minneapolis, MN; Mark Codner Plastic Surgery, Atlanta, GA

## Abstract

**Goals/Purpose:**

As inflation continues to rise, virtually all consumer markets are affected. However, the effect of these increases on aesthetic surgical procedures are not well defined. An important relational understanding of these effects can help aesthetic practices offer fair market prices to benefit both patients and practices alike. The purpose of this study was to quantify temporal changes in pricing for the most common aesthetic surgeries and to make comparisons to various indices of inflation and the stock market.

**Methods/Technique:**

Annual surgical fees for 18 aesthetic plastic surgery procedures were collected from 2007 to 2020 using the ASAPs and ASPS yearly plastic surgery statistic reports. In addition, annual consumer price indices (CPI) for all items, physicians’ services, and hospital services were collected from the U.S Bureau of Labor Statistics. Finally yearly Dow Jones Industrial (DJI) Averages were collected during the same time frame. Correlation statistics, generalized additive models, and parametric tests were performed to understand the relationships of these surgical costs to other indices.

**Results/Complications:**

A total of 322 annual measurements were collected for analysis. The cumulative percent change from 2007 to 2020 was highest for rhytidectomy, blepharoplasty, and otoplasty 38.1%, 33.4%, and 30.4%, respectively. Liposuction had the lowest overall cumulative percent change at -10.0%. Kendall correlation test revealed that out of all surgeries, male gynecomastia had the strongest correlation with time, Tau= 0.74, *p*=0.001. Breast augmentation, blepharoplasty, and abdominoplasty also correlated with time. When comparing the temporal change in prices to the various indices through nonparametric general additive modeling, breast augmentation, blepharoplasty, facelift, and liposuction costs changed at a statistically different rate than the overall CPI of all items, *p*=0.035, 0.018, 0.012, and <0.001, respectively. In contrast, rates of price changes in rhinoplasty, buttock lift, and breast lift surgery have not differed significantly from that of the CPI. The DJI has grown at rates far greater than the price of any aesthetic surgery procedure and models have been statistically different.

**Conclusion:**

While over the past decade aesthetic surgery costs have changed, certain procedure costs such as the rhytidectomy have greatly increased, while others have modestly increased, and liposuction has even decreased. Interestingly, the cost for the majority of the most performed aesthetic procedures are not greatly impacted by inflation. This suggests that other forces such as supply and demand may have a greater impact on price changes over the years. Many surgical procedures however were found to have similar rates of price change as the CPI, suggesting that these surgeries and their costs are more vulnerable to inflationary adjustments. Furthermore the price changes of aesthetic surgeries seem to be independent of how the stock market fluctuates and therefore possibly independent of other markets as a whole. This study has several limitations, with the data source as its greatest. The ASPS and ASAPs statistic reports are based on surveys from national members and computational adjustments, which have inherent biases and can vary significantly in any given year. Furthermore the various modeling and statistical analyses performed herein were based on several assumptions and estimates. An understanding of the price trends in aesthetic surgery over time and in relation to consumer price indices and the DJI can help plastic surgery practices and patients better appreciate the economics surrounding aesthetic surgeries.

